# Potential Biomarkers Associated with Prognosis and Trastuzumab Response in HER2+ Breast Cancer

**DOI:** 10.3390/cancers15174374

**Published:** 2023-09-01

**Authors:** Ana Carla Castro-Guijarro, Angel Matias Sanchez, Marina Inés Flamini

**Affiliations:** 1 Laboratorio de Biología Tumoral, Instituto de Medicina y Biología Experimental de Cuyo (IMBECU), Consejo Nacional de Investigaciones Científicas y Técnicas (CONICET), Universidad Nacional de Cuyo, M5500 Mendoza, Argentina; 2 Laboratorio de Transducción de Señales y Movimiento Celular, Instituto de Medicina y Biología Experimental de Cuyo (IMBECU), Consejo Nacional de Investigaciones Científicas y Técnicas (CONICET), Universidad Nacional de Cuyo, M5500 Mendoza, Argentina

**Keywords:** trastuzumab, T-DM1, lapatinib, metastasis, breast cancer

## Abstract

**Simple Summary:**

Breast cancer is the most common cancer among women worldwide. Overexpression of the HER2 receptor is associated with a worse prognosis and poorer survival. Consequently, several anti-HER2 therapies have been developed, such as trastuzumab. However, resistance still affects a significant population and is currently a major challenge in clinical oncology. Therefore, this study aims to identify biomarkers to predict disease progression and anticipate the efficacy of therapy to avoid therapeutic failure. We identified deregulated genes in trastuzumab-resistant cells associated with cell adhesion and migration. We demonstrate that combined anti-HER2 therapies are encouraging since low doses exhibit a synergism interaction and efficiently inhibit cell adhesion and migration critical process in cancer metastasis. We found deregulated proteins in resistant cells that may be potential biomarkers of response to therapy and may be involved in therapy resistance, useful to predict survival and response to trastuzumab therapy in HER2-positive breast cancer patients. These findings are promising for personalized breast cancer management to mitigate resistance and maximize the safety and efficacy of anti-HER2 therapies.

**Abstract:**

Breast cancer (BC) is the most common malignancy among women worldwide. Around 15–25% of BC overexpress the human epidermal growth factor receptor 2 (HER2), which is associated with a worse prognosis and shortened disease-free survival. Therefore, anti-HER2 therapies have been developed, such as monoclonal antibodies (trastuzumab, Tz), antibody–drug conjugates (ado-trastuzumab emtansine, T-DM1), and pharmacological inhibitors of tyrosine kinase activity (lapatinib, Lp). Although Tz, the standard treatment, has significantly improved the prognosis of patients, resistance still affects a significant population of women and is currently a major challenge in clinical oncology. Therefore, this study aims to identify potential biomarkers to predict disease progression (prognostic markers) and the efficacy of Tz treatment (predictive markers) in patients with HER2+ BC. We hypothesize that proteins involved in cell motility are implicated in Tz-resistance. We aim to identify alterations in Tz-resistant cells to guide more efficient oncologic decisions. By bioinformatics, we selected candidate proteins and determined how their expression, localization, and the process they modulate were affected by anti-HER2 treatments. Next, using HER2+ BC patients’ data, we assessed these proteins as prognostic and predictive biomarkers. Finally, using Tz-resistant cells, we evaluated their roles in Tz response. We identified deregulated genes associated with cell motility in Tz/T-DM1-resistant vs. -sensitive cells. We showed that Tz, T-DM1, and Lp decrease cell viability, and their effect is enhanced in combinations. We determined synergism between Tz/T-DM1 and Lp, making possible a dose reduction of each drug to achieve the same therapeutic effect. We found that combinations (Tz/T-DM1 + Lp) efficiently inhibit cell adhesion and migration. Furthermore, we demonstrated the induction of FAK nuclear and cortactin peri-nuclear localization after T-DM1, Lp, and Tz/T-DM1 + Lp treatments. In parallel, we observed that combined treatments downregulate proteins essential for metastatic dissemination, such as SRC, FAK, and paxillin. We found that low vinculin (VCL) and cortactin (CTTN) mRNA expression predicts favorable survival rates and has diagnostic value to discriminate between Tz-sensible and Tz-resistant HER2+ BC patients. Finally, we confirmed that vinculin and cortactin are overexpressed in Tz-resistance cells, SKBR3-RTz. Moreover, we found that Tz plus FAK/paxillin/cortactin-silencing reduced cell adhesion/migration capacity in Tz-sensitive and -resistant cells. In conclusion, we demonstrate that combined therapies are encouraging since low doses of Tz/T-DM1 + Lp inhibit metastatic processes by downregulating critical protein expression and affecting its subcellular localization. We propose that vinculin and cortactin might contribute to Tz-sensibility/resistance in BC cells. Finally, we identify potential prognostic and predictive biomarkers that are promising for personalized BC management that would allow efficient patient selection in order to mitigate resistance and maximize the safety and efficacy of anti-HER2 therapies.

## 1. Introduction

Breast cancer (BC) is the most common tumor disease diagnosed in women worldwide, with a high mortality incidence [1]. Mammary tumors are highly heterogeneous and are classified into five intrinsic subtypes based on gene expression profiling: luminal A, luminal B, HER2-enriched, basal-like, and normal-like. This classification is a clinically valuable tool for predicting prognosis and assigning treatments [2]. Approximately 15–25% of BCs are HER2-enriched, which means tumors overexpress the human epidermal growth factor receptor 2 (HER2, also known as ErbB2) on their surface. HER2+ BC patients exhibit an aggressive form of the disease associated with a poor prognosis characterized by shorter disease-free intervals and reduced survival [3,4].

HER2 is a tyrosine kinase receptor that belongs to the epidermal growth factor receptor family (HER), which also comprises EGFR (or HER1), HER3, and HER4. HER2 overexpression results in ligand-independent HER2/HER2 dimerization, leading to the constitutive activation of its signaling. In addition, HER2 dimerizes with the other members of the HER family, the HER2/HER3 heterodimer being the most oncogenic. Homo- or heterodimerization induces the activation of several downstream signaling pathways, including MAPK and PI3K/Akt, triggering uncontrolled cell proliferation, motility, and survival, supporting aberrant cell growth and tumor progression [5,6,7].

Since HER2 signaling predicts aggressive behavior in BC, HER2-targeted therapies have been developed [3,4]. The strategies currently used are monoclonal antibodies (trastuzumab (Tz), pertuzumab (Pz), margetuximab), antibody–drug conjugates (ado-trastuzumab emtansine (T-DM1), trastuzumab-deruxtecan (T-DXd)), and pharmacological inhibitors of tyrosine kinase activity (lapatinib (Lp), neratinib, tucatinib) [8]. In the present work, we mainly focused on Tz, T-DM1, and Lp.

Tz is a humanized monoclonal antibody that binds to the extracellular IV domain of the HER2 receptor, impairing its activation [4,9]. T-DM1 is an antibody–drug conjugate that combines Tz with a cytotoxic agent called emtansine (DM1), a microtubule polymerization inhibitor. In the conjugated form, DM1 is inactive and only fulfills its cytotoxic effect once internalized in the lysosome of the cancer cells [10,11]. Lapatinib (Lp) is an oral reversible tyrosine kinase inhibitor (TKI) that binds to the intracellular domain of EGFR and HER2, preventing their kinase activity and the subsequent activation of downstream pathways [12].

Trastuzumab (Tz) is the gold standard treatment for early and advanced HER2+ BC. Despite Tz clinical efficacy, a significant fraction of patients does not benefit from Tz therapy due to resistance events [4,13]. In patients, resistance is defined as a tolerance to pharmaceutical treatment, where cancer cells resist the effects of the drug and thereby grow and reform tumors, a process known as recurrence or relapse [14]. Resistance to the therapy could be either primary (de novo) or secondary (acquired). Primary resistance is characterized by an absence of response since the start of the treatment. In secondary resistance, patients initially responded satisfactorily to therapy, but eventually progressed, evolving to a more aggressive diseases with less probability of recovery [4,13].

The molecular mechanisms of Tz resistance include: (1) epitope masking that prevents HER2-Tz binding, (2) the constitutive activation of HER2 downstream signaling, PI3K/Akt, (3) the upregulation of alternative pathways, (4) immunosuppression that impairs Tz mechanism action based on antibody-dependent cellular cytotoxicity, (5) HER2 downregulation because of defects in endocytosis or genetic instability, and (6) over- and under-expression of modulators of cell cycle and apoptosis (cyclin E, p27, survivin). New mechanisms of Tz-associated resistance are continually being described [15,16]. Therefore, patients may exhibit several types of resistance, making their identification and follow-up difficult. Furthermore, Tz-treated patients can exhibit cardiac toxicity or other severe adverse effects, leading to the discontinuation or interruption of treatment [17,18].

Although oncology recommendations are subject to change with the advent of new drugs and research, the current gold standard treatment for anti-HER2 therapy in the metastatic setting involves a Tz-based approach. Other treatments are needed upon disease progression or toxicity, including T-DM1 and Lp. For detailed information, we suggest consulting international guidelines (www.esmo.org/guidelines) (accessed on 17 March 2023) [19,20,21,22,23].

When the disease progresses due to Tz resistances, it acquires a more aggressive behavior with a minor probability of recovery. Currently, no validated biomarkers can predict the benefit of each anti-HER2 approach to guide individual therapeutic decisions [24]. The main reason is the difficulty in determining which resistance mechanisms develop in each patient [13].

Interestingly, Tz-resistant cells have deregulated cellular functions related to cell death, metabolism, DNA damage response, cell cycle, transcription, differentiation, cell adhesion, and migration [25]. Resistance events and metastasis are more frequent in the advanced stages of the disease and are the leading cause of death in cancer patients [26]. The metastatic process involves sequential steps to achieve cancer spread, including invasion of the cell matrix and cell migration, intravasation into the circulatory or lymphatic system, extravasation to secondary tissue, and finally t5he adhesion and colonization of secondary sites [27]. Considering that adhesion and migration, critical events in cell metastasis, are altered in resistant cells, we wished to identify whether key proteins that regulate these processes are implicated in developing resistance to Tz therapy. We intend to identify alterations in Tz-resistant cells, particularly in deregulated genes/proteins that modulate these processes and could be used as prognostic and predictive biomarkers. We also propose to identify how the potential biomarker could be modified after the effects of anti-HER2 therapies, Tz, T-DM1, and Lp, alone or in combination. The present study aims to contribute knowledge that will allow the development of tools to efficiently select patients to mitigate resistance and maximize the safety and efficacy of anti-HER2 treatments.

## 2. Materials and Methods

### 2.1. Cell Culture and Treatments

The human breast carcinoma cell lines SKBR3 and BT-474 were obtained from the American Type Culture Collection (ATCC, Rockville, MD, USA). Cells were cultured in RPMI 1640 (Gibco, Rockville, MD, USA) supplemented with 10% fetal bovine serum (FBS), penicillin, and streptomycin (Sigma-Aldrich, St. Louis, MO, USA). Cell lines were maintained in a humidified atmosphere containing 5% CO_2_ at 37 °C. Both cells overexpress the HER2 (Neu/ErbB2) gene; SKBR3 is a widely used breast cancer model representing the HER2-enriched subtype (HER2+/ER−), and BT-474 corresponds to a luminal B (HER2+/ER+) experimental cell model.

*Spheroids (3D culture)* were generated using the liquid overlay technique. Briefly, 10,000 cells per well were seeded in 96-well U-bottom plates coated with 1% agarose and incubated at 37 °C in an incubator with 5% CO_2_ for 72 h until the formation of spheroids.

*Generation of acquired trastuzumab resistance model, SKBR3-RTz:* In vitro, the most common method for generating a drug-resistant cancer cell line involves repeated treatment of the parental cells with the anticancer drugs for an extended period [28]. We established the acquired trastuzumab-resistant model, SKBR3-RTz, by the treatment of parental cells, SKBR3, with trastuzumab for six months, starting with a dose of 10 μg/mL in the first month and increasing to 15 μg/mL for the other five months. We based this on the protocol followed by Díaz-Rodríguez et al. (2019) [25]. In vitro, cells are considered resistant when the treatment fails to inhibit survival, and thus, resistant cells proliferate at a similar rate to untreated control cells (parental cell line) [28]. We confirmed our acquired Tz resistance model by a proliferation/viability assay (MTT).

*Treatments:* The humanized monoclonal antibody trastuzumab (Tz, Herceptin^®^), pertuzumab (Pz, Perjeta^®^), and the antibody–drug conjugate ado-trastuzumab emtansine (T-DM1, Kadcyla^®^) were obtained from ROCHE S.A.Q.e I (Buenos Aires, Argentina) and dissolved in sterile water. The tyrosine kinase inhibitor lapatinib (Lp, Tykerb^®^) was obtained from Novartis (Basel, Switzerland) and was dissolved in DMSO. The doses selected for each anti-HER2 drug were taken from the existing literature. Generally, Tz and T-DM1 are used in a dose range of 10–20 μg/mL and Lp in a dose range of 0.3–2 μg/mL. We covered these concentrations in viability testing (MTT) using 0.1–100 μg/mL. In combinations, we tested concentrations with a constant ratio of 1 (Lp): 10 (Tz/T-DM1) based on other studies that evaluated the same combination [29,30,31]. It is important to state that in monoculture cell models, the effects of Tz and T-DM1 are underestimated since antibody-dependent cellular cytotoxicity, their primary mechanism of action, is not considered. Recombinant human HRG beta 1 protein was obtained from Abcam and was prepared in sterile apyrogenic water. All experiments were performed in triplicate, and representative images are shown.

### 2.2. Bioinformatics

*Differential gene expression analysis (DGE):* We used two microarray gene expression datasets to define transcriptomic modifications associated with Tz resistance (GSE119397 and GSE100192). They were programmatically downloaded from the publicly available Gene Expression Omnibus database (ncbi.nlm.nih.gov/geo/) (accessed on 23 May 2022) using the R GEOquery package [32]. The GSE119397 dataset consists of a model of in vitro resistance to trastuzumab (BT-474-RTz) with five repeats and their parentals BT-474 with eight repetitions. The GSE100192 dataset comprises T-DM1-resistant clones (BT-474-RT-DM1) with three replications of each and three replicates of the parental cell line BT-474. Differential gene expression analysis (DGE) was performed using the R *limma* package [33], comparing resistant vs. parental cells in each case. In all cases, log2 fold change values were obtained associated with exact *p*-values. We initially selected only those genes deregulated in the same way in both Tz and T-DM1 resistance models. This approach was taken because Tz and T-DM1 have similar structures and mechanisms of action. Thereby, it is to be expected that the molecular events that trigger resistance to these therapies are also similar. This strategy decreases the initial number of deregulated candidate genes in the Tz resistant phenotype.

*Identification of deregulated genes associated with cell motility:* The *PANTHER* classifications (www.pantherdb.org) (accessed on 1 June 2022) [34] based on the gene ontologies (GO) cell movement (GO: 0006928), actin filament-based process (GO: 0030029), and cell adhesion (GO: 0007155) were used to specifically determine the deregulated genes in Tz- and T-DM1-resistant cells that are involved in cell adhesion and migration process.

*Paxillin-interactors analysis:* Direct and functional paxillin-interactors were searched using the STRING database v11.5 (https://string-db.org) (accessed on 21 June 2022) [35]. Interaction scores > 0.4 were applied to construct the protein–protein interaction network.

### 2.3. Cell Viability

The viability was assessed using the MTT [3-(4,5-dimethylthiazol-2-yl)-2,5-diphenyltetrazol] assay. Monolayer cells or spheroids were seeded in 96-well plates at different concentrations of anti-HER2 therapies (Tz, T-DM1, and Lp) administrated as a single agent (0.1–100 μg/mL), or in combined treatments, Tz + Lp and T-DM1 + Lp (1:01–100:10 µg/mL). After 72 h, the medium was removed, and the cells were incubated with 0.5 mg/mL MTT (Sigma-Aldrich, St. Louis, MO, USA) for 4 h. Then, the MTT was removed, and the formazan crystal rings were dissolved with DMSO. Absorbance at 570 nm was measured using a microplate reader (MULTISKAN EX; Thermo Scientific). Cell viability was calculated as a percentage of viability in treated cells compared to untreated cells, as described by Castro-Guijarro et al. (2022) [36].

### 2.4. Analysis of Drug Interactions

CompuSyn was used to complement the results obtained in the cell viability assay. This software characterizes the pharmacological interaction between the drugs in the combined treatments (Tz + Lp and T-DM1 + Lp). CompuSyn utilizes the combination index (CI) to quantify synergism and antagonism in drug combinations based on the mass–action law designed by Chou and Talalay [37,38]. Synergism and antagonism were defined as a more- or a less-than-expected additive effect, respectively. The software also determines the dose-reduction index (DRI) that measures how much the dose of each drug, when combined, can be reduced to obtain a given biological result compared with the doses needed of each drug, without combination, for obtaining the same biological effect. Both indices are essential from a clinical standpoint where synergism and dose reduction may predict reduced toxicity toward the host while retaining therapeutic efficacy. CI, the affected fraction (FA), and dose-reduction index (DRI) values were calculated from the effects of varying doses on cell viability inhibition rates in the MTT assay. CompuSyn recommended a dose range that takes some doses above IC50, and some doses below (at least three points are necessary), for the construction of dose–effect curves used in CI and DRI determination. Software tolerates different units and concentration ratios between drugs. In constructing our curves, we met these requirements and obtained a sufficiently high r (>95) to arrive at reliable conclusions. CI = 1 denotes an additive effect, CI < 1 indicates synergism, and CI > 1 is antagonism. DRI = 1 means no dose reduction, DRI < 1 shows no favorable dose reduction, and DRI > 1 is favorable dose reduction. The affected fraction (Fa, range 0–1) was obtained via the following equation: 1—(% survival or unaffected fraction/100%), with 1 corresponding to 100% cell survival, as described by Vanderhoeven et al. (2018) [39].

### 2.5. Cell Adhesion Assay

Cells were exposed for 72 h to the mono drugs (1 μg/mL Tz, 1μg/mL Pz, 1 μg/mL T-DM1, 0.1 μg/mL Lp) and the combinations (1:0.1 μg/mL Tz + Lp, 1:0.1 μg/mL T-DM1 + Lp, 1:1 μg/mL Tz + Pz) with and without the transfection with specific siRNAs versus FAK, paxillin, and cortactin. After the treatments, cells were trypsinized and suspended in PBS containing trypan blue to determine the number of viable cells and seed the same viable cell density for each experimental condition. Briefly, 3 × 10^4^ SKBR3, 5 × 10^4^ BT-474, and 3 × 10^4^ SKBR3-RTz cells/well were seeded into 96-well plates previously coated with 1% sterile gelatin (Sigma-Aldrich). Cells were incubated at 37 °C for 2 h. Non-adherent cells were removed by gentle washing with PBS. The attached cells were fixed with 4% p-formaldehyde and stained with 10% ethanol/crystal violet for 20 min. Absorbance at 570 nm was measured using a microplate reader (MULTISKAN EX; Thermo Scientific, St. Leon-Rot, Germany). The absorbance is directly proportional to the number of adherent cells. The results are expressed as the percentage of cell adhesion, normalized with respect to control, which is considered 100%. Images were captured using a Nikon Eclipse E200 microscope coupled with a high-resolution CCD digital camera. The protocol was previously described in Castro-Guijarro et al. (2022) [36].

### 2.6. Wound Healing Assay

A scratch wound assay was conducted to assess the influences of the mono drugs (1 μg/mL Tz, 1 μg/mL Pz, 1 μg/mL T-DM1, 0.1 μg/mL Lp) and the combinations (1:0.1 μg/mL Tz + Lp, 1:0.1 μg/mL T-DM1 + Lp, 1:1 μg/mL Tz + Pz) for 72 h, with and without the transfection with specific siRNAs against FAK, paxillin, and cortactin. SKBR3 and SKBR3-RTz cells were seeded at the same density for each condition in 24-well plates and incubated until 70–80% confluence. Wounds were made in the monolayers by scratching the surface with a pipette tip (10 µL) as uniformly and straight as possible. The cells were washed, and the treatments were added. Cytosine β-D-arabinofuranoside hydrochloride (10 μM), an inhibitor of DNA strand separation that prevents cell division, was added to each well. Cell migration was monitored for 72 h. The migration distance was analyzed by phase-contrast microscopy, and closed areas were quantified using ImageJ software. Each wound measurement at 72 h was compared with the same photo and with the same place at 0 h, and the measures considered cell motility and not cell quantity or density. Cell migration was calculated as a percentage of migrated area in treated cells compared to untreated cells.

### 2.7. Spheroids Migration

Ten spheroids/well were collected and transferred to a 6-well plate containing the single treatments Tz (1 μg/mL), T-DM1 (1 μg/mL), or Lp (0.1 μg/mL) or the combined treatments Tz + Lp (1:0.1 μg/mL) or T-DM1 + Lp (1:0.1 μg/mL). Cytosine β-D-arabinofuranoside hydrochloride (10 μM) was added to each well. After 72 h, images were taken by phase-contrast microscopy. Cell migration was calculated by measuring the area of the halo of migration using ImageJ software and derived as the percentage of migrated area in treated cells compared to untreated cells.

### 2.8. Immunoblotting

Cells were harvested in lysis buffer including 100 mM Tris-HCl (pH 6.8), 4% SDS, 20% glycerol, 1 mM Na_3_VO_4_, 1 mM NaF, 1 mM PMSF, 1 mM PIC, and 1 mM PhiC. In fractionation experiments, after treatments, the cells were washed in PBS and resuspended in a lysis buffer for nuclear and cytoplasmic protein extraction, as reported by Andrews et al. (1991) [40]. Cell lysates were separated by SDS-PAGE and transferred into PVDF membranes. The primary antibodies used were: SRC (sc-5266), FAK (sc-271195-R), paxillin (sc-31010), cortactin (sc-11408), p-AKT^T308^ (sc-16646-R), p-mTOR^S2448^ (sc-101738) and actin (sc-1615) from Santa Cruz Biotechnology; HER2 (ab16901) from Abcam; vinculin (MAB3574) from Calbiochem; GAPDH (cat# 2118) from Cell Signaling, and HDAC1 (WH0003065M2) from Sigma-Aldrich. The secondary antibodies used were: anti-rabbit IgG-HRP (sc-2357), anti-mouse IgG-HRP (sc-358914), and anti-goat IgG-HRP (sc-2354) from Santa Cruz Biotechnology. Primary and secondary antibodies were incubated using standard techniques. Immunodetection was accomplished using enhanced chemiluminescence and was recorded with a quantitative digital imaging system (Chemidoc XRS with Image Lab, Bio-Rad, Hercules, CA, USA).

### 2.9. Transfection Experiments

The synthetic On-TARGETplus SMARTpool small interfering RNAs (siRNA) reagents against human FAK, and control siRNA (D-001810-01-05) were purchased from Dharmacon (Thermo Fisher Scientific Inc., Waltham, MA, USA). The siRNAs against paxillin and cortactin were purchased from Santa Cruz Biotechnology. SKBR3 and SKBR3-RTz (70–80% confluent) were incubated with 50–70 nM target siRNA or control siRNA for 48 h in serum-free media using Lipofectamine (Invitrogen, Carlsbad, CA, USA), as previously described [39,41]. Cells were treated after siRNA transfection. The efficacy of gene silencing was assessed by Western blot assay. Original blots can be found in Supplementary File S1.

### 2.10. Cell Immunofluorescence

SKBR3 and BT-474 cells were grown on coverslips and exposed to 1 μg/mL Tz, 1 μg/mL T-DM1, 0.1 μg/mL Lp, and their combinations Tz + Lp and T-DM1 + Lp (1:0.1 μg/mL) for 72 h. Cells were fixed with 4% p-formaldehyde for 30 min and permeabilized with 0.1% triton for 5 min. Blocking was performed with 3% bovine serum albumin for 30 min at room temperature. Then, the cells were incubated with FAK (BD 610088, Transduction Laboratories) or cortactin overnight at 4 °C. The cells were incubated with Alexa Fluor 488 (A-11001, Invitrogen) or Dylight 488 (DI-1488, Vector Laboratories) for 90 min at room temperature. Then, the cells were incubated with Texas Red-Phalloidin (TRPh, Sigma-Aldrich) for 30 min to visualize actin filaments. The nuclei were counterstained with 4′-6-diamidino-2-phenylindole (DAPI, Sigma-Aldrich), and coverslips were mounted with Vectashield mounting medium (Vector Laboratories). Immunofluorescence was visualized using a Nikon Eclipse E200 microscope and recorded with a high-resolution DP70 Olympus digital camera, as described by Mondaca et al. (2021) [6] and Castro-Guijarro et al. (2022) [36].

### 2.11. In Silico Analysis for Predictive Biomarkers

*KM plotter:* A prognostic biomarker predicts the evolution of disease (survival), independent of treatment. Thus, it enables the identification of patients with a more aggressive tumor. We used the platform Kaplan–Meier (KM) plotter (kmplot.com) to assess the correlation between the expression of our genes of interest (mRNA) and survival [42]. The platform used databases from the GEO, EGA, and TCGA repositories with the established inclusion criteria of untreated and treated patients with BC HER2+. We analyzed the prognostic values of genes using the following JetSet probes: 216836_s_at (ERBB2), 213324_at (SRC), 208820_at (PTK2), 201087_at (PXN), 200931_s_at (VCL), and 201059_at (CTTN) [43]. Patients were split into low- and high-expression groups, according to the median expression values, through auto-select best cut-off. The Kaplan–Meier method compared the two patient cohorts through relapse-free survival (RFS, *n* = 285) and distant metastasis-free survival (DMSF, *n* = 160) curves, and these were evaluated by the log-rank test. The force of prediction of all genes considered simultaneously as a genetic signature was also assessed.

*ROC plotter:* A predictive biomarker anticipates whether a particular treatment has a benefit. Thus, it helps to select the appropriate patients for a specific treatment over another. The predictive impacts of the expressions of our genes of interest (ERBB2, SRC, PTK2, PXN, VCL, and CTTN) were analyzed using the ROC plotter tool (rocplot.org) (accessed on 12 December 2022) [44]. The platform used databases from the GEO repository that met our inclusion criteria: patients with HER2+ BC treated with Tz. We used the identical probes mentioned above. Patients were assigned into responder or non-responder cohorts by auto-selecting the best cut-off. The response was assessed considering pathological complete response (pCR, *n* = 26 including 17 responders and 9 non-responders) or relapse-free survival at 5 years (RFS, *n* = 24 including 12 responders and 12 non-responders). Genes were evaluated individually and as a genetic signature. The area under the curve (AUC) determined the prognostic power of each gene. AUC > 0.6 indicates potential clinical utility as a cancer biomarker.

### 2.12. Statistical Analysis

Statistical analysis was performed with one-way analysis of variance (ANOVA) followed by Tukey–Kramer Multiple-Comparisons or Kruskal–Wallis tests using GraphPad Prism 5.03 software. *p* < 0.05 was considered statistically significant. All values were expressed as mean ± standard error (SD) of three independent experiments. DGE bioinformatics analyses were performed using R version 4.0.4 in a Windows environment with Intel Core i7 with 32 GB of RAM.

## 3. Results

### 3.1. Transcriptomic Modifications Associated with Tz and T-DM1resistance

**Figure 1:**
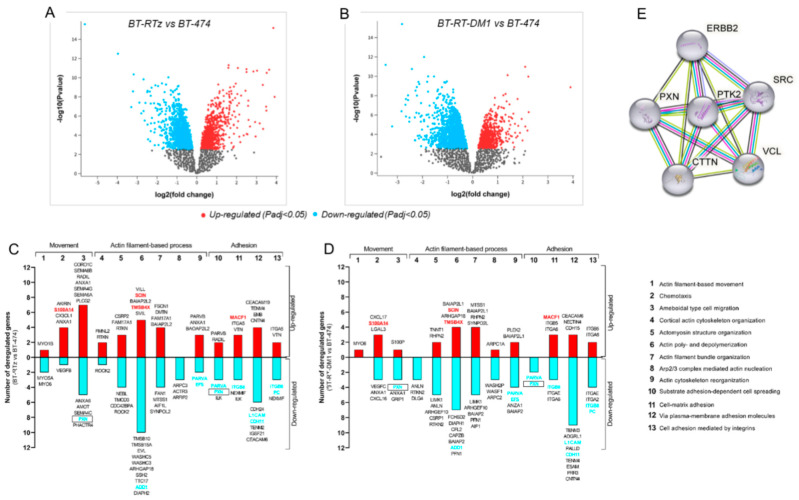
Differential gene expression involved in the metastatic process in BC cells resistant to Tz or T-DM1. Differential gene expression volcano plots were obtained using *AnalyzeGeo* (https://www.ncbi.nlm.nih.gov/geo/geo2r/) (accessed on 25 May 2022) On the *x*-axis, the log2 fold change of (**A**) BT-RTz vs. BT-474, and (**B**) BT-RT-DM1 vs. BT-474. Genes upregulated (*in red*) or downregulated (*in light blue*) are shown (*p* < 0.05). Gray dots represent genes with no statistical significance. Gene ontology characterization by the PANTHER tool of genes downregulated or upregulated in (**C**) BT-RTz and (**D**) BT-RT-DM1 vs. control. The genes analyzed are those involved in different processes related to movement, adhesion, and metastasis. *Red* columns represent the number of upregulated genes, and *light blue* indicates the number of downregulated genes. Those genes that were equally deregulated in both resistant models are highlighted in *red or blue*. Deregulated genes simultaneously involved in two different processes in both resistant cell models are remarked in a rectangle. (**E**) The STRING tool was queried to establish paxillin interactors. Nodes represent proteins. Colored lines between the proteins indicate the type of interaction evidence used in predicting the associations. Red: fusion evidence; green: neighborhood evidence; blue: co-occurrence evidence; purple: experimental evidence; yellow: text-mining evidence; light blue: database evidence; black: co-expression evidence.

To identify potential transcriptomic differences responsible for Tz resistance, a dataset consisting of a Tz-resistant cell model (BT-RTz) and the respective parental cells (BT-474) was used, and a differential gene expression (DGE) analysis were performed. We observed 3148 genes that were deregulated, including 1965 downregulated and 1183 upregulated (Figure 1A). However, it is expected that many of these genes are not related to Tz resistance. Therefore, to refine our study, we performed a new DGE analysis, this time using a dataset consisting of T-DM1-resistant cells (BT-RT-DM1) and their parental cells (BT-474). This approach was used because Tz and T-DM1 have similar structures and mechanisms of action; thus, the molecular events that trigger resistance to these therapies are expected to be similar. We consider in the subsequent analysis only those genes that are deregulated in the same way in both resistant models. Similarly, 2794 genes were differentially expressed in BT-RT-DM1 vs. BT-474; 1873 were downregulated and 921 were upregulated (Figure 1B). More details about the DGEs analyses can be found in supplemental data online (Figure S1).

Next, we intend to identify alterations in Tz-resistant cells that could be used as prognostic and predictive biomarkers. The contribution of bioinformatics databases and the scarce information related to metastatic processes made it attractive and novel for us to focus our study on identifying biomarkers associated with these altered functions in resistant cells. Thus, we used the PANTHER classification to identify deregulated genes in Tz- and T-DM1-resistant cells, focusing on movement, actin-filaments-based processes, and adhesion, critical events for metastasis, where Tz resistance is widely reported. We found that PXN (paxillin), ADD1 (α-adducin), PARVA (α-parvin), EFS (embryonal fyn-associated substrate), ITGB8 (integrin subunit beta), L1CAM (neural cell adhesion molecule 1), CDH11 (cadherin-11), and PC (pyruvate carboxylase) were downregulated in both resistant models (marked in light blue in Figure 1C,D, and shown in supplemental data online (Figure S1)). Meanwhile, S100A14 (S100-A14 protein), SCIN (scinderin), TMSB4X (thymosin β4), and MACF1 (microtubule actin crosslinking factor 1) were upregulated (marked in red in Figure 1C,D, and in supplemental data online (Figure S1)). These results suggest that actin dynamics, adhesion, and migration would be altered in Tz and T-DM1-resistant cells (Figure 1C,D). Furthermore, we observed that PXN (encoding paxilin) simultaneously modulates movement (ameboidal type cell migration) and adhesion (substrate adhesion-dependent cell spreading), being a candidate for our study (marked with a rectangle, Figure 1C,D).

Then, we reasoned that if paxillin is deregulated in the Tz- and T-DM1-resistance cells model, it is likely that its direct and functional interactors are also affected. Therefore, we studied its interaction network using STRING software. We limited the analysis to ten proteins, focusing on those genes/proteins involved in cell adhesion and migration. We observed that paxillin (PXN) interacts with HER2 (ERBB2), SRC (SRC), FAK (PTK2), vinculin (VCL), and cortactin (CTTN), making this group of genes/proteins our set of interest for the following analyses (Figure 1E).

### 3.2. Effect of Anti-HER2 Therapies on Cell Viability

**Figure 2:**
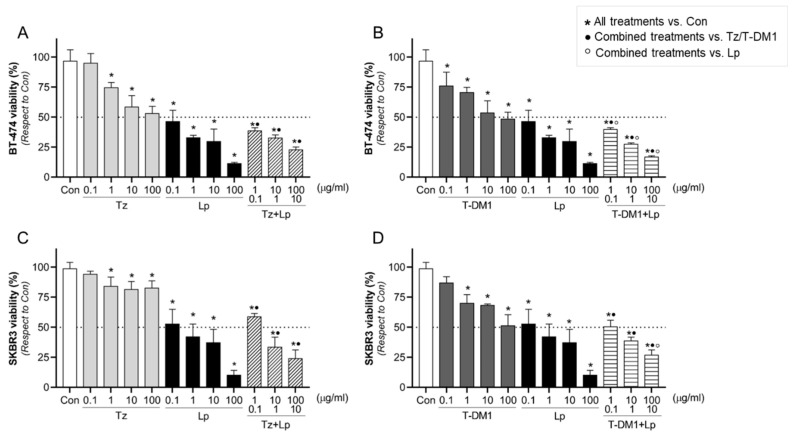
BT-474 and SKBR3 viability after anti-HER2 treatments. Cell viability was evaluated by MTT assay in (**A**,**B**) BT-474 and (**C**,**D**) SKBR3 BC cells. Cells were treated with increasing doses of Tz (0.1–100 μg/mL), T-DM1 (0.1–100 μg/mL), Lp (0.1–100 µg/mL), and their combination Tz + Lp in a constant relation of 10:1 µg/mL or T-DM1 + Lp in a constant relation of 10:1 µg/mL for 72 h. The results are expressed as a percentage (%) of surviving cells compared to the control (Con, 100% survival). The dotted line in the *y*-axis represents IC50 values for each condition. * = *p* < 0.05 vs. control, • = *p* < 0.05 in combined treatments vs. Tz/T-DM1, and ° = *p* < 0.05 in combined treatments vs. Lp. All experiments were performed in triplicate; representative images are shown.

The use of combination therapy is a strategy to treat drug-resistant cancers. To identify how the potential biomarkers will be modified after the anti-HER2 therapies, we examined different doses of the monoclonal antibody (Tz) or antibody–drug conjugate (T-DM1) in combination with a tyrosine kinase inhibitor (Lp) on their antitumor efficacy in BT-474 and SKBR3 cells. In BT-474 cells, we found that all treatments except the lowest concentration of Tz (0.1 μg/mL) inhibited cell viability in a dose-dependent manner (Figure 2A,B). In SKBR3 cells, all mono drugs suppressed cell viability except the lowest concentrations of Tz and T-DM1 (0.1 μg/mL) (Figure 2C,D). To analyze whether the efficacy of the drugs is greater alone or in combination, we compared the effects of combinations versus single drugs (supplemental data online Figure S2), considering the same concentrations as were used in the combined treatment. In both cells, we observed that the inhibitory effect of Lp alone was always higher than Tz or T-DM1 alone, but not with respect to the combinations (Tz/T-DM1 + Lp) (Figure 2A–D, and supplemental data online (Figure S2)). Although Tz and T-DM1 as single agents slightly reduced cell viability, their combination with Lp produced a significant inhibition (Figure 2A–D, and supplemental data online (Figure S2)). While our results show that Lp’s inhibitory effects perform better than any other option, Lp in vivo failed to demonstrate the same efficacy [45]. Taking these into account and considering that in resistant tumors, combination therapies are the approach of preference, we selected for subsequent experiments 1 μg/mL of Tz/T-DM1 plus 0.1 μg/m of Lp, which is the minimum dose of combinations that achieve a considerable inhibitory effect.

### 3.3. Identification of Synergistic Drug Combinations

**Figure 3:**
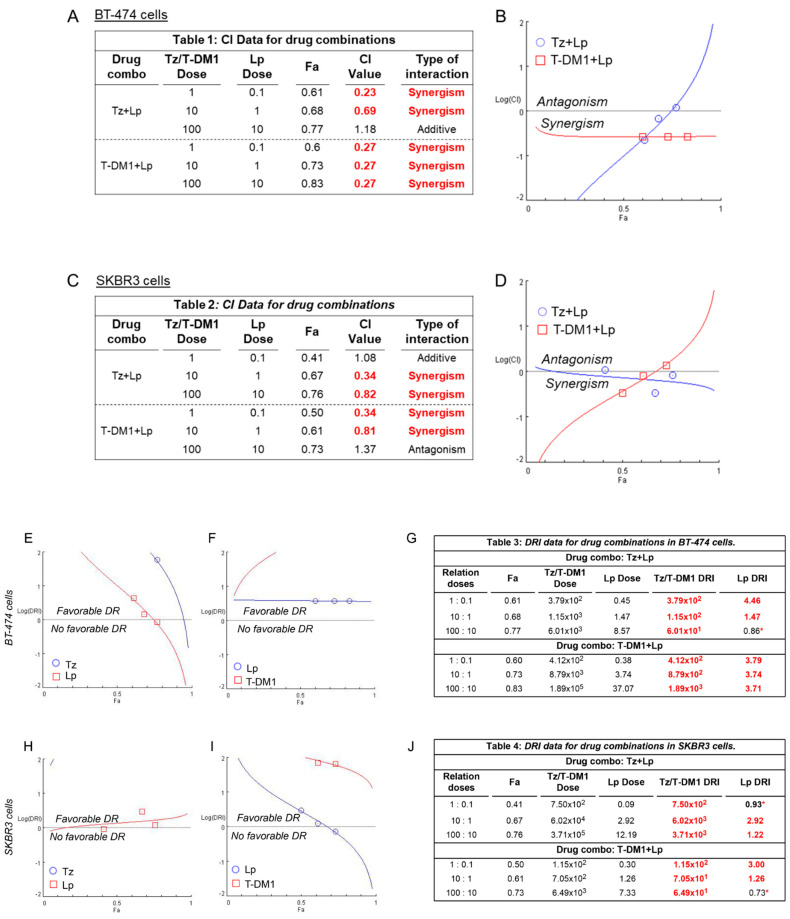
Pharmacological interaction analysis between Tz, T-DM1, and Lp in HER2+ BC cells. CompuSyn software was used to characterize the pharmacological interaction produced by the combined treatments with Tz (1–100 μg/mL) plus Lp (0.1–10 μg/mL) or T-DM1 (1–100 μg/mL) plus Lp (0.1–10 μg/mL) in (**A**,**B**) BT-474 and (**C**,**D**) SKBR3 cells. Combination index (CI) and affected fraction (Fa) levels were calculated from the effects of varying doses of anti-HER2 drugs on cell viability inhibition rates in the MTT assay. CI = 1 denotes an additive effect, CI < 1 indicates synergism, and CI > 1 is antagonism. The dose reduction index (DRI) is represented for each drug in (**E**,**H**) Tz + Lp and (**F**,**I**) T-DM1 + Lp combinations for both BC cell models. Quantitative values are displayed in the tables for (**G**) BT-474 and (**J**) SKBR3 cells. DRI = 1 denotes no dose reduction, DRI < 1 indicates non-favorable dose reduction (marked with an asterisk *), and DRI > 1 is favorable (*marked in red*). All doses are expressed in μg/mL.

To complement the previous results, we used data from the MTT assay to quantitatively characterize the pharmacological interaction between the drugs in the combined treatments (Tz + Lp and T-DM1 + Lp) in SKBR3 and BT-474 cells by Compusyn [37,38]. In BT-474 cells, the combination index (CI) values for doses 1:0.1 μg/mL and 10:1 μg/mL of the Tz + Lp combination were 0.23 and 0.69, respectively, indicating a synergistic interaction (highlighted in red, Figure 3A). Meanwhile, the CI value for the highest dose (100:10 μg/mL) was 1.18, associated with an additive effect (Figure 3A,B). In the T-DM1 + Lp combined treatment, all CI values observed were 0.27, showing synergistic action (highlighted in red, Figure 3A,B).

In SKBR3 cells, Tz plus Lp was additive at the lower concentration of 1:0.1 μg/mL (CI = 1.08) and synergistic at 10:1 μg/mL and 100:10 μg/mL (CI = 0.34 and 0.82, respectively) (Figure 3C,D). In addition, CI values for the T-DM1 + Lp combination showed synergism for the doses 1:0.1 μg/mL and 10:1 μg/mL (CI = 0.34 and 0.81, respectively). In contrast, drugs combined at the highest dose (100:10 μg/mL) manifested an antagonistic effect (CI = 1.37) (Figure 3C,D).

On the other hand, we also determined the dose reduction index (DRI) to evaluate whether it is possible to reduce the concentration of each drug in the combinations to achieve the same therapeutic action versus when they are administered as mono drugs. In both cell lines, we observed that all doses of Tz and T-DM1 allow a favorable dose reduction (DRI > 1 marked in red, Figure 3E–J). The reduction in Lp dose is possible only when combined with 1–10 µg/mL of Tz and 1–100 µg/mL of T-DM1 in BT-474 BC cells (DRI >1 marked in red, Figure 3E,G). In SKBR3 cells, a reduction in Lp dose is possible when it is administrated with 10–100 µg/mL of Tz and 1–10 µg/mL of T-DM1 (DRI >1 marked in red, Figure 3H,J). Furthermore, the software performs a simulation that returns the predicted CI and DRI values for the 5–97% affected fraction range (Supplemental Data Online Figures S3–S6).

### 3.4. Adhesion and Migration Inhibition with Anti-HER2 Therapies

**Figure 4:**
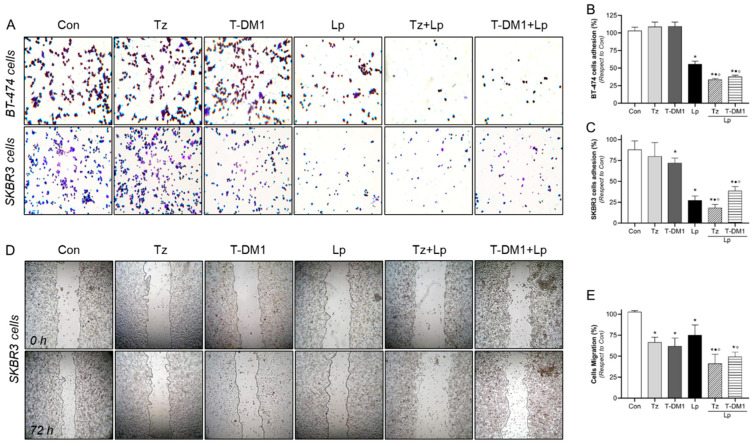
Tz/T-DM1 plus Lp inhibit cell adhesion and migration. HER2+ BC cells were treated with Tz (1 μg/mL), T-DM1 (1 μg/mL), Lp (0.1 µg/mL) or their combinations for 72 h, and cell adhesion and migration assays were performed. (**A**–**C**) Representative images and percentages of cells adhered to gelatin, and (**D**,**E**) representative images and percentages of SKBR3 cell migration. Gap closure was quantified by measuring areas using the ImageJ software. Adhesion/migration results are expressed as a percentage of attached/migrated cells vs. control cells. * = *p* < 0.05 vs. control, • = *p* < 0.05 in combined treatments vs. Tz/T-DM1, and ° = *p* < 0.05 in combined treatments vs. Lp. All experiments were performed in triplicate; representative images are shown.

As a first approach to determining a promising marker of prognosis and response to anti-HER2 therapies, we explored the actions of 1 μg/mL Tz, 1 μg/mL T-DM1, 0.1 μg/mL Lp and their combinations (1:0.1 μg/mL Tz/T-DM1 + Lp) for 72 h in those cellular processes that are deregulated in resistant cells, such as cell adhesion and migration. In BT-474 cells, we found that Tz and T-DM1 had no effect. However, Lp induced adhesion inhibition by approximately 44% (Figure 4A,B). Adding Lp to Tz/T-DM1 treatment resulted in a 62–66% reduction in adherence, suggesting that Lp improves the Tz/T-DM1 effect (Figure 4A,B).

In SKBR3 cells, we visualized that Tz did not affect cell adhesion. Likewise, T-DM1, Lp, Tz + Lp, and T-DM1 + Lp efficiently inhibited cell adhesion by 28%, 73%, 82%, and 61%, respectively. Again, the administration of Lp to Tz/T-DM1 induced a more powerful adherence inhibition than Tz/T-DM1 alone (Figure 4A,C).

Since BT-474 has reduced migratory capability, we evaluated the process of cell migration in an appropriate model, such as SKBR3 cells. The wound healing assays showed that treatments based on mono drugs (Tz, T-DM1, and Lp) significantly diminished migration by 25–38%. However, the combined treatments (Tz + Lp, T-DM1 + Lp) produced inhibition by 51–59%. Furthermore, a synergistic effect was evidenced in the Tz + Lp condition (Figure 4D,E).

Since Tz and pertuzumab (Pz) is a well-tolerated and highly effective treatment in HER2+ BC, we also evaluate the effects of 1 μg/mL Tz, 1 μg/mL Pz, and their combination (Tz + Pz, 1:1 μg/mL) on SKBR3 cells’ motility for 72 h. Cell adhesion was only slightly reduced by the combination Tz + Pz. Migration was inhibited by Tz and Pz as mono drugs; however, a major inhibition was achieved by the combination Tz + Pz (Supplemental Data Online Figure S7).

### 3.5. Inhibitory Action of Anti-HER2 Therapies on BC Spheroids 3D Model

**Figure 5:**
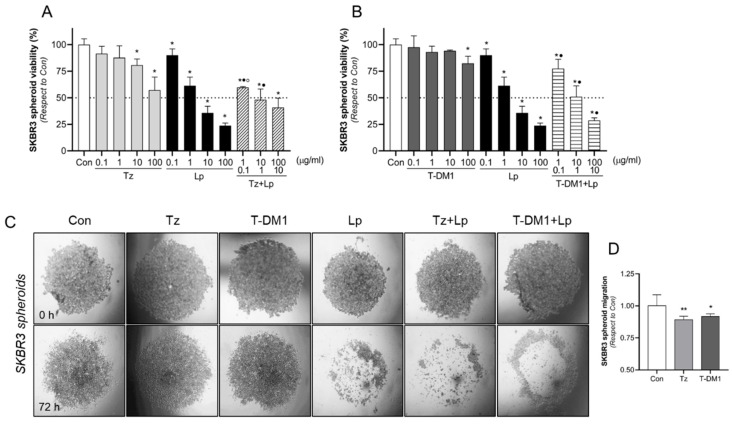
Effects of HER2 therapies on cell viability and motility through spheroids 3D model. The viability assay was evaluated by the MTT assay in SKBR3 spheroids. Spheroids were treated with increasing doses of (**A**) Tz (0.1–100 μg/mL), Lp (0.1–100 μg/mL), and their combination Tz + Lp in a constant relation (10:1 µg/mL) or (**B**) T-DM1 (0.1–100 μg/mL), Lp (0.1–100 μg/mL), and their combination T-DM1 + Lp in a constant relation of 10:1 µg/mL during 72 h. The results are expressed as a percentage (%) of surviving cells compared to the control (Con, 100% survival). The dotted line in the *y*-axis represents the IC50 values for each condition. * = *p* < 0.05 vs. control, • = *p* < 0.05 in combined treatments vs. Tz/T-DM1, and ° = *p* < 0.05 in combined treatments vs. Lp. All experiments were performed in triplicate; representative images are shown. (**C**,**D**) Representative images of the cell migration halo in the presence of Tz (10 μg/mL), T-DM1 (10 μg/mL), Lp (1 µg/mL), or their combinations for 72 h. Migration was calculated as the difference between migration (halo) areas at 72 h vs. 0 h using the ImageJ software v1.54f and presented as the percentage of migrated cells vs. control cells. * = *p* < 0.05 vs. control; ** = *p* < 0.01 vs. control.

Since spheroids mimic the architecture of tumors and drug penetration more effectively than monolayer cells, we tested the effects of anti-HER2 therapies on cell viability and migration in SKBR3 spheroids. Spheroids were treated with Tz (0.1–100 μg/mL), T-DM1 (0.1–100 μg/mL), Lp (1–10 μg/mL), and their combination for 72 h. We found that Tz decreased cell viability in doses higher than 10 μg/mL, while T-DM1 did so only at the higher concentration tested (100 μg/mL). Lp diminished cell viability in all concentrations (Figure 5A,B). Only 10 and 100 μg/mL of Lp and 10:1 and 100:10 μg/mL of combined treatments achieved the half-maximal inhibition (IC50) (Figure 5A,B). Once again, the addition of Lp to Tz/T-DM1 produced a significant reduction in cell viability compared to the same concentration of Tz/T-DM1 alone (Figure 5A,B). We noted that it is necessary to use higher doses in a 3D model than in a 2D model (Figure 2A–D) to achieve a similar inhibitory effect.

Considering these results, we have evaluated migration capacity using higher doses than 2D culture. After 72 h of the treatment with Tz (10 μg/mL), T-DM1 (10 μg/mL), Lp (1 μg/mL), and their combinations, we found that Tz and T-DM1 exert a slight inhibitory effect, decreasing spheroid migration by 11% and 8%, respectively (Figure 5C,D). On the contrary, Lp and the combinations Tz + Lp and T-DM1 + Lp achieved complete inhibition, since no migration halo was identified (Figure 5C,D).

### 3.6. Anti-HER2 Treatments Control Proteins Involved in Cell Motility

**Figure 6:**
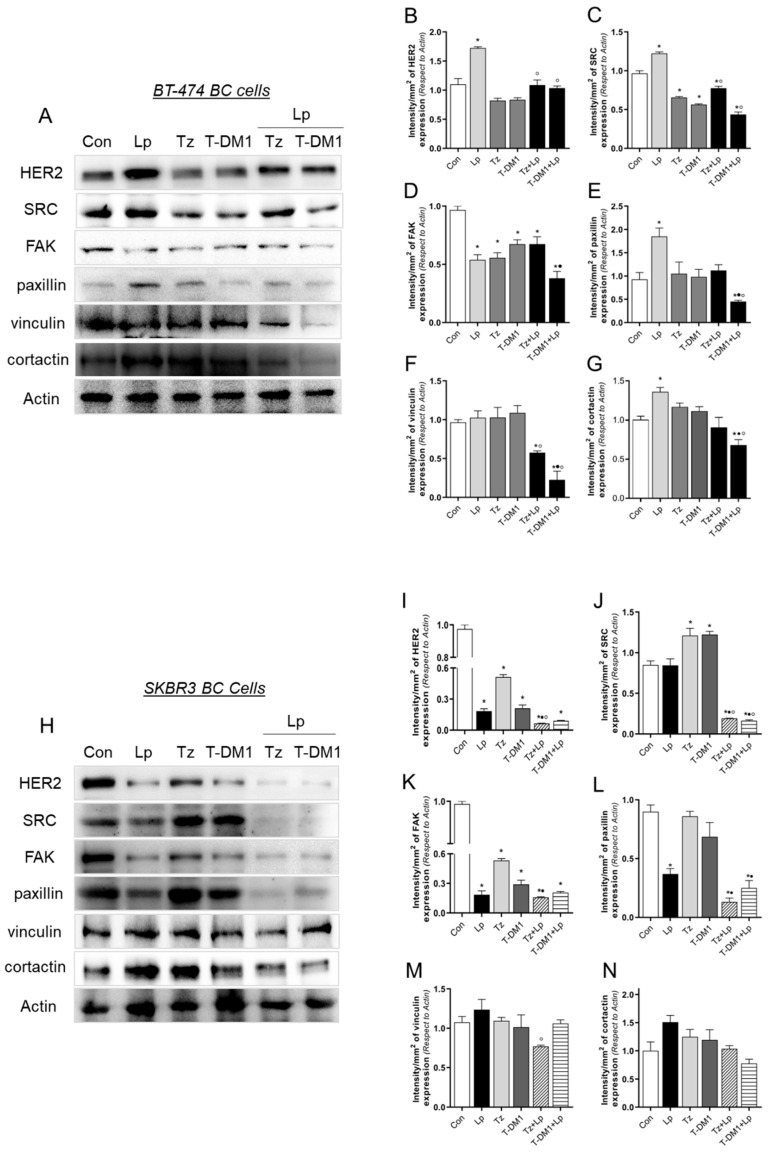
Expression of paxillin interactors after anti-HER2 therapies. (**A**) BT-474 cells were exposed to Tz (1 μg/mL), T-DM1 (1 μg/mL), Lp (0.1 µg/mL) or their combinations for 72 h, and the expressions of HER2, SRC, FAK, paxillin, vinculin and cortactin were analyzed by Western blot assay. Actin expression is shown as a loading control. (**B**–**G**) Densitometric quantifications of HER2, SRC, FAK, paxillin, vinculin, and cortactin bands. Intensity values were adjusted to the corresponding intensity values of actin and then normalized to the control. (**H**) SKBR3 cells were exposed to Tz (1 μg/mL), T-DM1 (1 μg/mL), Lp (0.1 µg/mL) or their combinations for 72 h, and the pattern expressions of HER2, SRC, FAK, paxillin, vinculin, and cortactin were analyzed by Western blot assay. Actin expression is shown as a loading control. (**I**–**N**) Densitometric quantifications of HER2, SRC, FAK, paxillin, vinculin, and cortactin bands. Intensity values were adjusted to the corresponding intensity values of actin and then normalized to the control. * = *p* < 0.05 vs. control, • = *p* < 0.05 in combined treatments vs. Tz/T-DM1, and ° = *p* < 0.05 in combined treatments vs. Lp. All experiments were performed in triplicate; representative images are shown.

Since our in silico studies demonstrated that paxillin, an essential protein in metastatic events, is deregulated in Tz- and T-DM1-resistant cells (Figure 1A–D), and that the STRING tool determines proteins that interact with paxillin (Figure 1E), we next assessed the expression of paxillin interactors after anti-HER2 therapies. We performed treatments with Tz (1 μg/mL), T-DM1 (1 μg/mL), Lp (0.1 μg/mL), and their combinations for 72 h. In BT-474 cells, Lp treatment increased the expressions of HER2, SRC, paxillin, and cortactin (Figure 6A–C,E,G). Treatment with Tz and T-DM1 induced the significant downregulation of SRC and FAK (Figure 6A,C,D). When Tz and T-DM1 were combined with Lp, the downregulation of SRC and FAK persisted. Additionally, paxillin, vinculin, and cortactin expression were downregulated in combinations (Figure 6A,E–G). These results suggest that Lp, as a mono drug, is ineffective due to its inducing of the expression of oncogenic proteins. Regarding combination treatments, T-DM1 + Lp is more potent than Tz + Lp, and combinations are more efficient in downregulating critical proteins involved in metastatic processes in BT-474 cells.

In SKBR3 cells, the treatment with Lp decreased HER2, FAK, and paxillin expression (Figure 6H,I,K,L). It is important to note that we observed a contrary effect after the Lp treatment between BT-474 and SKBR3 BC cells (Figure 6A,H). In addition, Tz/T-DM1 caused the downregulation of HER2 and FAK (Figure 6H–J). Combined treatments enhanced the inhibitory effects of downregulating HER2, SRC, FAK, and paxillin expression (Figure 6H–L). As in BT-474 cells, these results suggest that the combinations Tz + Lp and T-DM1 + Lp are more effective in inhibiting the expression of proteins involved in metastasis than mono drugs.

### 3.7. Anti-HER2 Therapies Induce Nuclear FAK and Perinuclear Cortactin Localization

**Figure 7:**
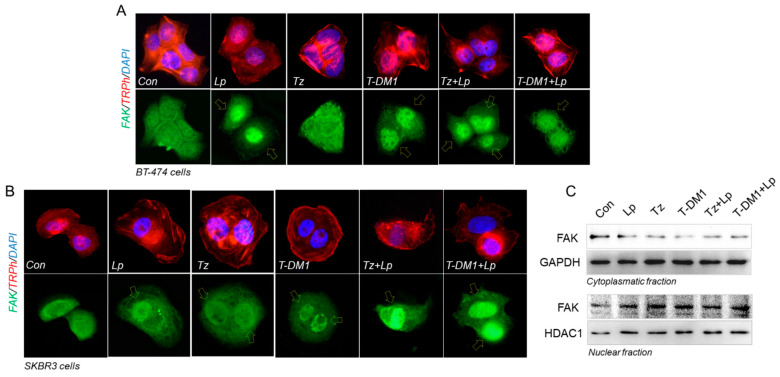

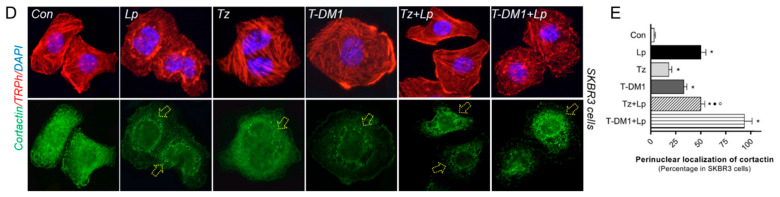
Redistribution of FAK and cortactin subcellular localization after anti-HER2 treatments. After the administration with Tz (1 μg/mL), T-DM1 (1 μg/mL), Lp (0.1 µg/mL) or their combinations for 72 h, (**A**) BT-474 and (**B**) SKBR3 cells were subjected to an immunofluorescence assay vs. anti-FAK linked to Alexa Fluor 488 (green), filamentous actin was stained with phalloidin linked to Texas Red (red), and nuclei were counterstained with DAPI (blue). Yellow arrows indicate nuclear FAK relocalization. Representative images are shown. SKBR3 cells were treated with Tz (1 μg/mL), T-DM1 (1 μg/mL), Lp (0.1 µg/mL) or their combinations for 72 h. (**C**) Cell fractionation assay and Western blot analyses for FAK were performed. As the loading control for the cytoplasmic fraction, we used GAPDH, and for the nuclear fraction we used HDAC1. (**D**) Immunofluorescence assay was performed, staining with anti-cortactin linked to Dylight 488 (green), filamentous actin was stained with phalloidin linked to Texas Red (red), and nuclei were counterstained with DAPI (blue). Yellow arrows indicate nuclear perinuclear cortactin redistribution. (**E**) Quantification of perinuclear cortactin in the different conditions, normalized with respect to the control. Perinuclear-localized cortactin was counted in 40 different cells. * = *p* < 0.05 vs. control, • = *p* < 0.05 in combined treatments vs. Tz/T-DM1, and ° = *p* < 0.05 in combined treatments vs. Lp. All experiments were performed in triplicate; representative images are shown.

We next performed an immunofluorescence assay staining FAK (an indicator of focal adhesion sites) and cortactin (regulator of actin nucleation), two proteins upstream and downstream of paxillin, respectively. We treated BT-474 and SKBR3 cells with Tz (1 μg/mL), T-DM1 (1 μg/mL), Lp (0.1 μg/mL) and their combination for 72 h to reveal FAK and cortactin subcellular localization. In BT-474 cells, in control and Tz treatment, FAK was homogeneously distributed between cytoplasm and nucleus. The exposure to Lp, T-DM1, Tz + Lp, and T-DM1 + Lp triggered nuclear FAK translocation (Figure 7A, *yellow arrows*).

SKBR3 cells were more affected than BT-474 since all treatments induced a nuclear FAK translocation (Figure 7B, *yellow arrows*). To confirm FAK’s subcellular localization, we used a nucleus/cytoplasm fractionation assay. We determined that all treatments induce a decrease in cytoplasmic fraction and an increase in nuclear FAK localization (Figure 7C).

By immunofluorescence, we visualized cortactin homogeneously diffusing throughout the cytoplasm and nucleus in control SKBR3 cells. The exposure of Lp, Tz, and T-DM1 as single agents induced cortactin perinuclear localization, and in combinations (Tz + Lp and T-DM1 + Lp) this effect was enhanced (Figure 7D,E).

### 3.8. VCL and CTTN mRNA Expression as a Prognostic Marker in HER2+ BC Patients

**Figure 8:**
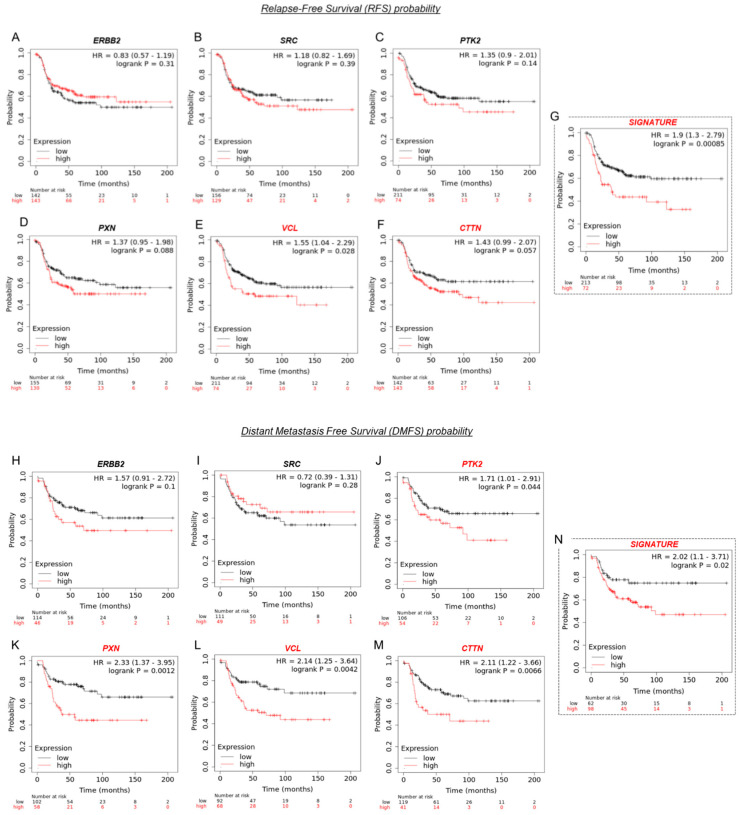
Expressions of genes involved in metastasis and survival in HER2+ BC patients. Kaplan–Meier survival curves for the HER2-positive BC cohort according to tumor expression of (**A**) ERBB2, (**B**) SRC, (**C**) PTK2, (**D**) PXN, (**E**) VCL, (**F**) CTTN and (**G**) the mean expression of the six biomarkers simultaneously as a genetic signature in terms of relapse-free survival (RFS) probability. Kaplan–Meier survival curves for the HER2-positive BC cohort according to tumor expression of (**H**) ERBB2, (**I**) SRC, (**J**) PTK2, (**K**) PXN, (**L**) VCL, (**M**) CTTN and (**N**) the mean expressions of the six biomarkers simultaneously as a genetic signature in terms of distant metastasis-free survival (DMFS) probability. Gene expression was segregated into low (blue) and high (red) expression according to the median expression values through auto-select best cut-off. Log-rank *p* values, hazard ratios (HR), and 95% confidence intervals are shown. Expressions of genes with a *p* < 0.05 are highlighted in red.

A prognostic biomarker predicts the evolution of disease (survival), independent of treatment, allowing the identification of patients with a more aggressive tumor. To analyze the prognostic values of our genes of interest (ERBB2, SRC, PTK2, PXN, VCL, and CTTN) in HER2+ BC patients, we used the KM plotter (Figure 8A–F). We observed that patients with low levels of VCL (vinculin, *Hazard Ratio* HR = 1.55; *p* = 0.028) and CTTN (cortactin, HR = 1.43; *p* = 0.057) mRNA expression have improved relapse-free survival (RFS) (Figure 8E,F *marked in red*), while ERBB2, SRC, PTK2, and PXN mRNA expression did not affect RFS probability (Figure 8A–D). When the expressions of all mRNAs (ERBB2, SRC, PTK2, PXN, VCL, and CTTN) were analyzed simultaneously as a genetic signature, the prognostic strength was greater (HR = 1.9; *p* = 0.00085) (Figure 8G, *marked in red*).

In parallel, we also analyzed the prognostic values of mRNA expression to predict distant metastasis-free survival (DMFS) rates (Figure 8H–M). Patients with high mRNA expression levels of PTK2 (FAK, HR = 1.71; *p* = 0.044), PXN (paxillin, HR = 2.33; *p* = 0.012), VCL (HR = 2.14; *p* = 0.0042), and CTTN (HR = 2.11; *p* = 0.0066) had significantly worse DMFS compared to the group of patients with low expression (Figure 8J–M, *marked in red*). ERBB2 and SRC mRNA expression did not affect DMFS probability (Figure 8H–I). When we analyzed the expression of all mRNAs (ERBB2, SRC, PTK2, PXN, VCL, and CTTN) as a genetic signature, we found that although it has prognostic force, the statistical significance was higher when PTK2, PXN, VCL, and CTTN were analyzed separately (Figure 8N).

### 3.9. VCL and CTTN mRNA as a Predictive Marker between Tz-Responder and Tz-Non-Responder HER2+ BC Patients

**Figure 9:**
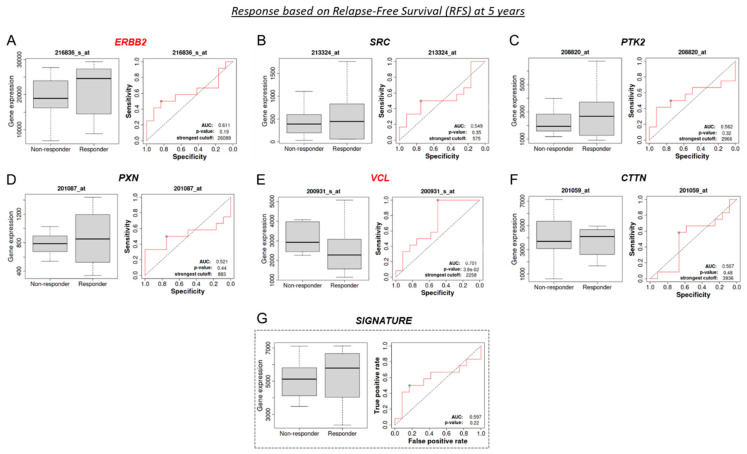

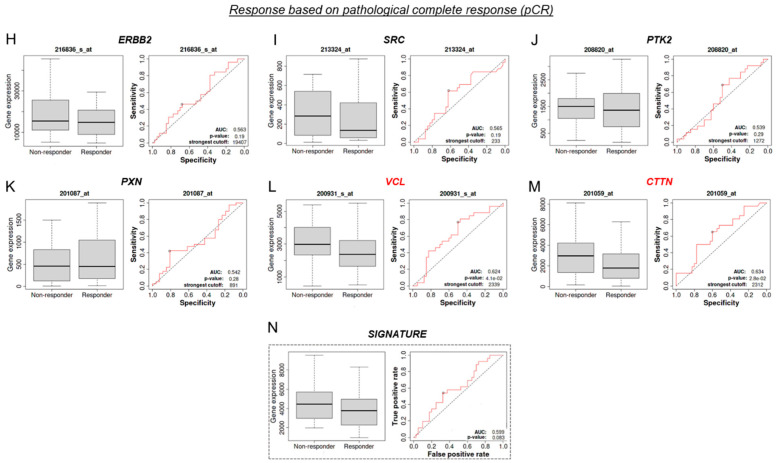
Predictive biomarkers of Tz response in HER2+ BC patients. Boxplots illustrating gene expression between Tz-responders and -non-responders in HER2+ BC patients and the respective receiver operating characteristic (ROC) curve. Boxplots and ROC curves for (**A**) ERBB2, (**B**) SCR, (**C**) PTK2, (**D**) PXN, (**E**) VCL, (**F**) CTTN, and (**G**) the six biomarkers simultaneously analyzed as a genetic signature, tested in terms of response based on relapse-free survival (RFS) at 5 years. Boxplots and ROC curves for (**H**) ERBB2, (**I**) SCR, (**J**) PTK2, (**K**) PXN, (**L**) VCL, (**M**) CTTN and (**N**) the six biomarkers simultaneously analyzed as a genetic signature, tested as predictive biomarkers in terms of response based on pathological complete response (pCR). An area under the curve (AUC) higher than 0.6 indicates that gene expression possesses a great discriminatory capability for predictive response to Tz treatment. The expressions of genes with an AUC > 0.6 are highlighted in red.

A predictive biomarker anticipates if there is a benefit or not of a particular treatment. Thus, it helps to select the appropriate patients for a specific treatment over another. To validate our genes of interest as potential predictive biomarkers of response to Tz therapy in HER2+ BC patients, we used HER2 BC patients’ data and the receiver operating characteristic (ROC) curves. In Figure 9, we assess the predictive impacts of ERBB2, SRC, PTK2, PXN, VCL, and CTTN in response to Tz therapy. The area under the curve (AUC) determined the prognostic power of each gene, where AUC > 0.6 indicates potential clinical utility as a cancer biomarker. Next, we noted different gene expressions of ERBB2 and VCL between Tz-responder and Tz-non-responder HER2+ patients. Based on RFS, we observed that a low gene expression of ERBB2 (AUC = 0.611; *p* = 0.19) was significantly associated with a lack of therapeutic response (Figure 9A, *marked in red*). Meanwhile, a low gene expression of VCL (AUC = 0.701; *p* = 0.038) was significantly associated with a favorable Tz response (Figure 9E, *marked in red*). SRC, PTK2, PXN, and CTTN expression did not affect the Tz response (Figure 9B–D,F). We found no predictive therapeutic response when we analyze the expressions of all our genes of interest as a genetic signature (Figure 9G).

In parallel, based on the pathological complete response (pCR, Figure 9H–M), we noted that low VCL (AUC = 0.624, *p* = 0.041) and CTTN (AUC = 0.634, *p* = 0.028) expression correlated with a favorable Tz response (Figure 9L–M, *marked in red*). In contrast, the gene expressions of ERBB2, SRC, PTK2, and PXN had no diagnostic efficacy in determining Tz response or in use as a genetic signature (Figure 9H–K,N).

### 3.10. Vinculin and Cortactin Are Involved in Tz Resistance in SKBR3-RTz

**Figure 10:**
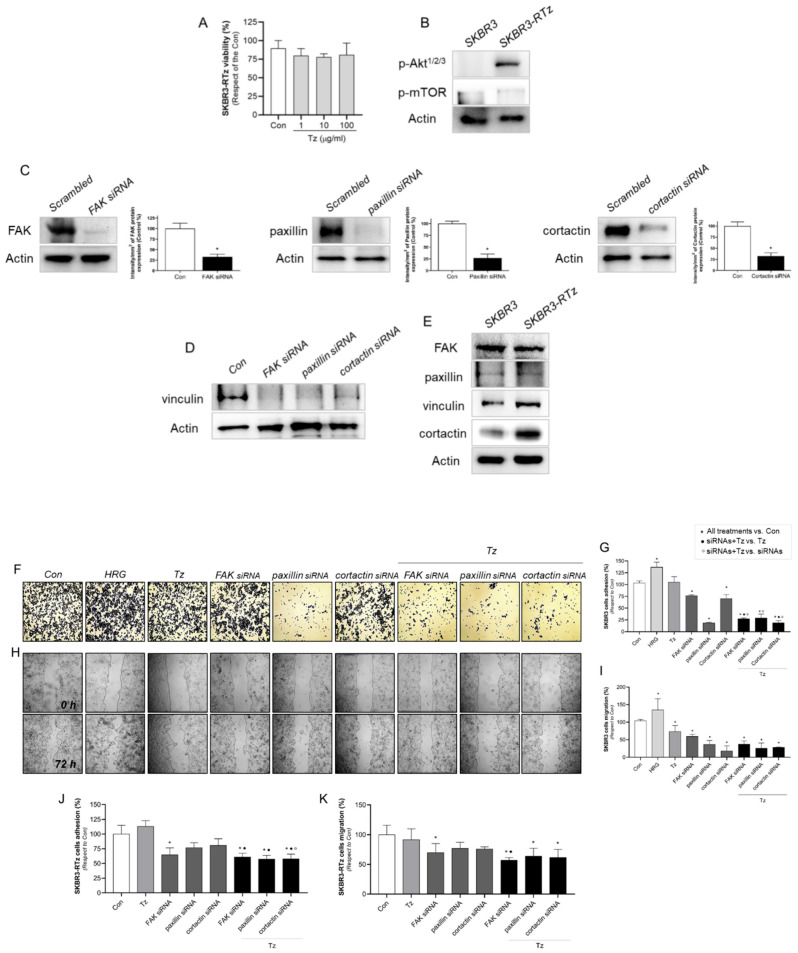
Vinculin and cortactin used as predictive biomarkers for Tz-sensibility/resistance. (**A**) Cell resistance was evaluated by MTT assay in SKBR3-RTz BC cells. Cells were treated with increasing doses of Tz (1–100 μg/mL) for 72 h. The results are expressed as a percentage (%) of surviving cells compared to the control (Con, 100% survival). (**B**) Extracts of SKBR3 and SKBR3-RTz cells were lysated and the expression levels of phospho-AKT^1/2/3^ and phospho-mTOR were analyzed by wWstern blot. (**C**) Cells were transfected with specific siRNAs vs. FAK, paxillin, cortactin or siRNA control (Scrambled). The efficacy of gene silencing was assessed by Western blot assay. (**D**) Regulation of vinculin expression by FAK, paxillin and cortactin, after specific siRNAs treatment. (**E**) SKBR3 and SKBR3-RTz cells were lysated and FAK, paxillin, vinculin, and cortactin expression were analyzed by Western blot assay. Actin expression is shown as a loading control. SKBR3 and SKBR3-RTz BC cells were treated with HRG (1 nM), Tz (1 µg/mL), siRNAs vs. FAK/paxillin/cortactin, combined or not with Tz (1 μg/mL) for 72 h, and (**F**,**G**,**J**) cell adhesion and (**H**,**I**,**K**) migration assay was performed. Adhesion/migration results are expressed as a percentage of attached/migrated cells vs. control cells. Representative images and percentages of cells adhered and migrated are shown. * = comparison between all treatments vs. Con, • = comparison between siRNAs + Tz vs. Tz, and ° = comparison between siRNAs + Tz vs. siRNAs. *p* < 0.05 was considered statistically significant. All experiments were performed in triplicate.

To support our findings, we established a model of acquired resistance, SKBR3-RTz, by sustained Tz exposure in parental cells. Resistance was evidenced by an MTT assay, where cell viability was not affected by 1–100 μg/mL of Tz during 72 h (Figure 10A). We analyzed two proteins that are linked to Tz resistance, such as Akt and mTOR, downstream effectors of the PI3K pathway. We observed that the phosphorylation of AKT and mTOR is increased in SKBR3-RTz compared to parental cells (Figure 10B).

Then, SKBR3 and SKBR3-RTz cells were transfected with FAK, paxillin, cortactin, or control siRNAs to assess their adhesion and migration ability. The silencing success was confirmed by Western blots, where we evidenced that siRNAs treatment inhibits target protein expression (Figure 10C). In addition, we demonstrated a dependence of vinculin expression on FAK/paxillin/cortactin. When cells were exposed to different siRNAs, vinculin was affected, decreasing the expression levels (Figure 10D).

In order to determine whether our potential biomarkers (FAK, paxillin, vinculin, and cortactin) are upregulated in resistant cells, we compared their expressions between SKBR3 and SKBR3-RTz cells. We determined that Tz- sensitive and -resistant cells express comparable levels of FAK and paxillin (Figure 10E). Meanwhile, vinculin and cortactin are overexpressed in SKBR3-RTz cells (Figure 10E). These results suggest that vinculin and cortactin might contribute to Tz- sensibility/resistance in BC cells.

Finally, in parental SKBR3 cells, FAK, paxillin, and cortactin silencing reduced cell adhesion. The treatment of Tz plus FAK/paxillin/cortactin siRNAs resulted in a potentiated adherence reduction (Figure 10F,G). In the wound healing assay, we observed that all treatments inhibit cell migration (Figure 10H,I). As a positive control, we used heregulin (1 nM, HRG), a selective ligand that triggers HER2 activity by binding HER3 and HER4 receptors and preferentially inducing HER2/HER3 heterodimerization [6]. HRG increased both adhesion and migration in SKBR3 cells (Figure 10F–I).

On the contrary, in SKBR3-RTz cells, we observed that Tz did not affect either adhesion or migration, showing that the cells are resistant to Tz. Only siRNA vs. FAK transfected cells showed reduced cell adhesion and migration (Figure 10I,J). However, when siRNAs vs. FAK, paxillin and cortactin were combined with Tz, all treatments diminished adhesion and migration with respect to control.

## 4. Discussion

Even though Tz is the gold standard for HER2+ BC patients and has dramatically improved their outcomes, most patients with advanced disease develop resistance and relapse by largely unknown mechanisms [4,13]. Furthermore, responding patients eventually experience toxicities [17,18]. However, no biomarkers predict patients who may benefit from or develop resistance [24]. Given the immense therapeutic benefits that the existence of markers would bring to distinguishing the most aggressive tumors and differentiating between responders and non-responders to a specific therapy, we attempt to identify potential biomarkers associated with prognosis and response to Tz in HER2+ BC.

Using bioinformatics data, we identified genes that are simultaneously deregulated in BT-474-RTz and BT-474-RT-DM1 resistance models vs. parental cells. Deregulated processes, including cell death, metabolism, DNA damage response, cell cycle, transcription, differentiation, adhesion and migration have been previously reported in Tz-resistant cells [25]. In this work, we focus on the study of deregulated genes critical for metastasis development, since this is the stage of the disease whereat resistance events are more frequent. Therefore, we analyzed the actin filaments-based process, movement, and adhesion. We found PXN, ADD1, PARVA, EFS, ITGB8, L1CAM, CDH11, PC, S100A14, SCIN, TMSB4X, and MACF1 mRNAs differentially expressed in resistant versus parental cells. In this sense, several authors have reported that paxillin, α-adducin, α-parvin, and cadherin-11 are altered in various types of cancer, including BC [39,46,47,48]. More importantly, paxillin has been reported as a determinant for Tz resistance [49]. Paxillin is a scaffold protein that regulates actin cytoskeleton dynamics and thereby cell adhesion and the migration of cancer cells [6,50]. Others and we have previously demonstrated its critical role in the spread of cancer and suggested it as a promising target for BC treatment [50,51].

In parallel, we evaluated the effects of Tz, T-DM1, Lp, and their combinations (Tz + Lp, T-DM1+ Lp) in HER2+ BC, BT-474 and SKBR3 cells. Consistent with other reports [52,53], we observed that Tz, T-DM1, and Lp decreased cell viability in both cell lines. It has been evidenced that the mechanism of cell viability inhibition by anti-HER2 drugs results from cell cycle arrest and increased apoptosis [54,55,56]. We noted that Tz and T-DM1, administered as a single agent, produced minimal inhibition, but their combination with Lp resulted in a potentiated effect. However, Tz/T-DM1 action may be underestimated since our in vitro model does not allow us to consider their main mechanisms of action in relation to antibody-dependent cellular cytotoxicity (ADCC). The enhanced inhibitory effect of Tz/T-DM1 plus Lp is due to the synergistic drug interaction, evidenced by CI values < 1. The synergistic relation between Tz/T-DM1 and Lp is related to the simultaneous inhibition of the oncogenic receptors HER2 and EGFR. Scaltriti et al. (2009) proposed that Lp potentiates the effects of Tz by inhibiting HER2 phosphorylation and reducing receptor degradation. Thus, Lp induces the accumulation of inactive HER2 dimers, increasing Tz binding and actions [57].

Although our study does not include toxicity assays, we found DRI values > 1 in combined treatments, which means favorable dose reduction for combined drugs is possible. DRI values > 1 are promising as they may allow for broadening the narrow therapeutic index or obtaining a better benefit/toxicity ratio, which is essential in cancer therapies associated with clinically relevant toxicities [17,18]. In this sense, Po-Hung Hsieh et al. (2022) demonstrated that low doses in appropriate patients may meaningfully reduce toxicity [58]. In any case, more research is needed to arrive at robust conclusions on this issue.

In addition, we found that Tz/T-DM1 combined with Lp is more effective than drugs alone in inhibiting adhesion and migration, critical processes associated with aggressiveness and metastatic potential in BC. Tz and T-DM1 as adhesion and migration inhibitors have been extensively demonstrated in HER2-overexpressing BC cells [6,39,59]. However, little is known of their effects as combined treatments.

Intratumor heterogeneity could be involved in drug resistance by affecting drug penetration, internalization, and efficacy [47]. Since 3D cell culturing permits mimicking the heterogeneity of in vivo tumors [48], we evaluated the efficacy of anti-HER2 therapies in SKBR3 cell spheroids. We also evidenced the superiority of the combined treatments in inhibiting viability and migration in 3D model. Nonetheless, higher doses were required to achieve significant inhibition than in the 2D model. In the same way, Boyer et al. (2021) [46] and Gangadhara et al. (2016) [60] found that cells in 3D culture lost sensitivity to Tz and T-DM1 compared to monolayer cells. We also determined that Tz and T-DM1 weakly decreased spheroids migration, whereas Lp and combinations (Tz + Lp and T-DM1 + Lp) achieved complete inhibition.

Overall, the results suggest that co-targeting HER2 using drugs that block the HER2 receptor extracellularly (Tz/T-DM1) and intracellularly (Lp) allows the better impairment of its signaling, and may be a mechanism to enhance drug activity and decrease resistance events. However, clinically, the superiority of Tz + Lp vs. Tz alone has not been conclusively determined. For instance, the NEOALTTO trial demonstrated that Tz + Lp significantly improved pCR rates compared with either drug alone [61]. However, the ALTTO trial showed no marked differences among Tz + Lp, Tz, and Lp groups regarding disease-free survival (DFS) [45]. Interestingly, Yuan et al. (2022) performed a meta-analysis of thirteen randomized controlled trials comparing Tz + Lp versus monotherapy. In line with our results, they conclude that Tz + Lp therapy is superior to Tz therapy alone concerning overall survival, DFS, pCR, and recurrence-free survival [23].

Our next step was to evaluate the effects of anti-HER2 therapies on paxillin and its interactors. In BT-474, we found that Lp induced the over-expression of several proteins related to the HER2 pathway [6], including HER2, SRC, paxillin, and cortactin. In this regard, Scaltriti, M., et al. (2009) demonstrated that Lp leads to a marked accumulation of inactive HER2 at the cell surface [57]. However, in SKBR3, we observed the opposite effect as Lp produced a down-regulation of HER2, FAK, and paxillin. Tz and T-DM1 also down-regulated HER2 expression only in SKBR3. The different mode of regulation of HER2 between both cells may be because we used a low dose that only affected the most sensitive SKBR3 cells. Several authors report HER2 downregulation with doses > 10 μg/mL Tz/T-DM1 [39,62]. Regarding combined treatments, HER2 expression is not affected in BT-474 cells, probably due to the compensation of inhibitory effects of Tz/T-DM1 and inducing effects of Lp. These discrepancies observed between BT-474 and SKBR3 cells could be attributable to the different basal expressions of HERs, Estrogen Receptor (crosstalk), and proteins linked to cell motility [6,63]. On the other hand, in both cells, Tz and T-DM1 inhibited FAK expression, consistent with other reports [39,64]. An essential finding of this work was that the combined treatments enhance the downregulation of proteins linked to metastasis in both cell lines.

Since the activity of proteins also depends on their cellular location, we analyzed FAK and cortactin expression by immunofluorescence. FAK is a marker of the early stages of cell motility, and cortactin is a controller of the later stages [65,66]. We found that Lp, T-DM1, and combined treatments induce nuclear FAK translocation in BT-474 and SKBR3 cells. FAK redistribution from the cytoplasm to the nucleus in response to stress agents has been previously reported by our group and others [36,39,67,68]. In addition, anti-HER2 treatments increased perinuclear cortactin, most highly in combined therapies. For cell motility, the recruitment of FAK and cortactin to focal adhesions (FAs) is necessary to induce actin rearrangement to assemble structures involved in cell migration and invasion. In any case, the absence of FAK in sites related to the formation of focal adhesions or cortactin in the actin reorganization reduces the chances of cell movement. Therefore, we suggest that the combinations are more potent, decreasing the expression and redistribution of pivot proteins and altering adhesive and migratory processes.

We used bioinformatics tools to determine the utility of our proteins of interest as potential biomarkers. Regarding prognostic markers, we found that low vinculin and cortactin mRNA expression predicts favorable survival rates in terms of relapse-free survival (RFS). As a genetic signature, the analysis of ERBB2, SRC, PTK2, PXN, VCL, and CTTN mRNA expression together has better prognostic value than they do individually. Furthermore, we found that low PTK2, PXN, VCL, and CTTN mRNA expression predicts favorable survival rates regarding distant metastasis-free survival (DMFS), also as a genetic signature. Not surprisingly, the low expression of FAK, paxillin, vinculin, and cortactin predicts good survival outcomes, considering that several studies report an association between their overexpression and tumor aggressiveness [50,69,70].

Regarding predictive markers, based on RFS, we demonstrated that high levels of ERBB2 and low VCL mRNA expression are associated with a favorable response to Tz. Meanwhile, low VCL and CTTN mRNA levels correlate to a favorable pathological complete response (pCR). These results are promising as no validated biomarker, apart from HER2, can predict the benefit of the Tz approach, and thus guide individual therapeutic decisions [24]. Other authors have established a relation between cortactin and vinculin overexpression and cancer aggression and resistance, and have also proposed them as promising prognostic and predictive biomarkers [71,72,73]. These proteins could be related as a study linked them, wherein vinculin mediated the action of cortactin [74].

Finally, we established an acquired Tz resistance model, SKBR3-RTz, to support our findings, and evaluated FAK, paxillin, vinculin, and cortactin involvement in Tz response. We determined that vinculin and cortactin were upregulated in resistance SKBR3-RTz cells, as expected regarding the results from the KM- and ROC- plotter. We determined that in SKBR3 cells, Tz and siRNAs vs. FAK/paxillin/cortactin decrease cell adhesion and migration, and the combination Tz + siRNAs potentiates the inhibition increasing Tz response. On the contrary, in SKBR3-RTz, Tz does not affect cell motility. Meanwhile, the specific siRNAs combined with Tz reduce adhesion and migration, weakening Tz resistance. Overall, these results suggest that vinculin and cortactin could contribute to Tz response.

In conclusion, we demonstrated that the combined therapies (Tz/T-DM1 + Lp) are promising because they offer a complete blockade of HER2 signaling due to their different mechanisms of action, improving cell viability, adhesion, and migration inhibition in low doses. Moreover, Tz/T-DM1 + Lp would efficiently inhibit metastatic processes by down-regulating and affecting the cellular localization of essential proteins. Furthermore, we identified vinculin and cortactin as potential prognostic and predictive biomarkers, which is promising for personalized BC management. We also proposed that vinculin and cortactin could contribute to Tz resistance. Further research into and monitoring of the real-time expression of these potential biomarkers could help prevent resistance events.

## Data Availability

The original contributions presented in the study are included in the article/Supplementary Material. Further inquiries can be directed to the corresponding authors.

## Supplementary Materials

The following supporting information can be downloaded at: https://www.mdpi.com/article/10.3390/cancers15174374/s1. Figure S1: Deregulated genes involved in the metastasis process in Tz and T-DM1 resistant cells vs. sensitive cells; Figure S2: Media values of raw data from the MTT assays; Figure S3: CI data for the drugs combinations in BT-474 cells; Figure S4: CI data for the drugs combinations in SKBR3 cells; Figure S5: DRI data for the drugs combinations in BT-474 cells; Figure S6: DRI data for the drugs combinations in SKBR3 cells; Figure S7: Effect of pertuzumab plus trastuzumab in SKBR3 adhesion and migration; File S1: Original blots.
